# Circular RNA PUM1 performs as a competing endogenous RNA of microRNA-340-5p to mediate DEAD-box helicase 5 to mitigate cerebral ischemia-reperfusion injury

**DOI:** 10.1080/21655979.2022.2068923

**Published:** 2022-05-05

**Authors:** Teng Hu, Di Li, TiePing Fan, XuSheng Zhao, ZhongJun Chen

**Affiliations:** Department of Neurological Intervention, Dalian Municipal Central Hospital, Dalian City, China

**Keywords:** Circular RNA PUM1, MicroRNA-340-5p, DDX5, cerebral ischemia-reperfusion nerve injury

## Abstract

Cerebral ischemia-reperfusion damages local brain tissue and impairs brain function, but its specific pathogenesis is still uncertain. Recent studies have clarified circPUM1 is aberrantly elevated in cerebral ischemia-reperfusion injury; however, circPUM1ʹs function in cerebral ischemia-reperfusion-induced neuronal injury remains ambiguous. The results illustrated circPUM1 and DEAD-box helicase 5 were decreased, but microRNA-340-5p was elevated in transient middle cerebral artery occlusion mice and oxygen glucose deprivation/reoxygenation-treated SH-SY5Y cells. Knockdown of circPUM1 aggravated the neuronal injury in transient middle cerebral artery occlusion mice and motivated glial cell activation, neuronal apoptosis and inflammation. Enhancing circPUM1 restrained oxygen glucose deprivation/reoxygenation-induced SH-SY5Y cell apoptosis, the release of lactate dehydrogenase and inflammatory factors, and activation of nuclear factor-kappaB pathway, while elevating microRNA-340-5p aggravated oxygen glucose deprivation/reoxygenation-induced cell damage. Functional rescue experiments exhibited that the impacts of knockdown or enhancement of circPUM1 were turned around by microRNA-340-5p downregulation and DEAD-box helicase 5 silencing, respectively. Moreover, it was demonstrated that circPUM1 competitively adsorbed microRNA-340-5p to mediate DEAD-box helicase 5. All in all, this study clarifies that circPUM1 mitigates cerebral ischemia-reperfusion-induced neuronal injury by targeting the microRNA-340-5p/DEAD-box helicase 5 axis.

## Graphical abstract

**Figure:**
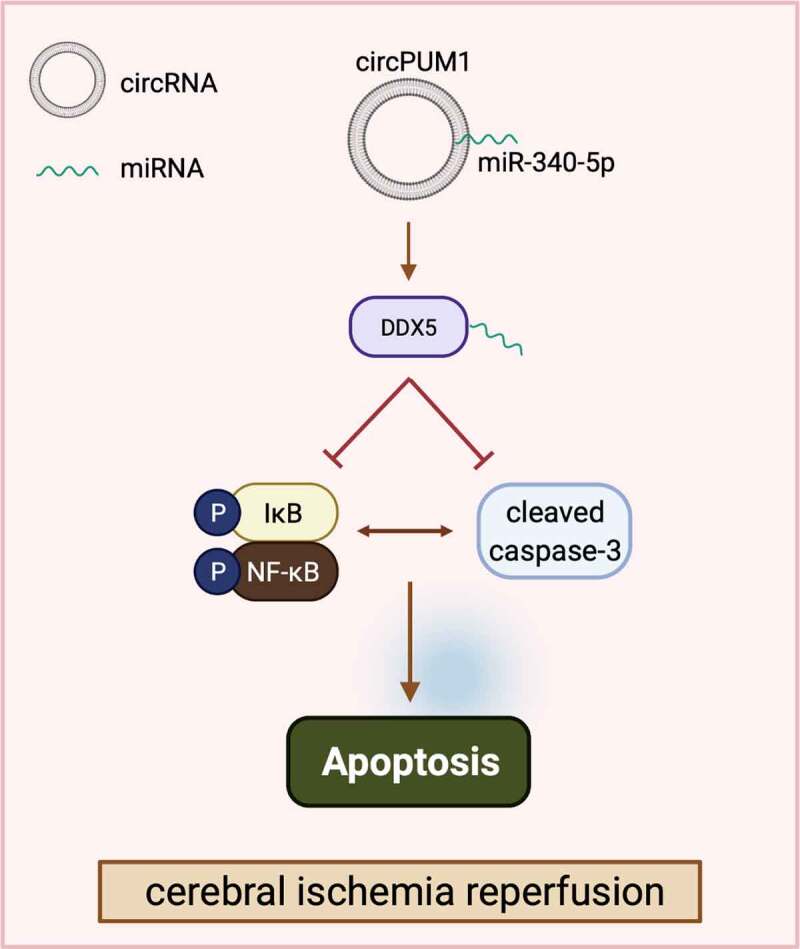

## Highlights

- circPUM1 and DDX5 expression decreases while miR-340-5p expression increases in CI/R injury.
- Knockdown of circPUM1 or overexpression of miR-340-5p aggravates CI/R injury.
- CircPUM1 competitively adsorbs miR-340-5p
- miR-340-5p mediates DDX5 expression
- CircPUM1 ameliorates CI/R-induced neuronal injury by regulating the miR-340-5p/DDX5 axis.

## 1. Introduction

Ischemic stroke is the second most frequent reason for death worldwide [1]. Cerebral ischemia-reperfusion (CIR) injury is induced by the restoration of blood flow after an ischemic stroke and can result in severe brain tissue damage [2]. Plentiful studies have clarified CIR injury covers multiple physiological and pathological processes, like inflammation, neuronal damage and oxidative stress, which in turn results in apoptosis and necrosis [3,4]. Fortunately, CIR-induced neuronal injury can be recovered by timely treatment [5]. However, the specific molecular mechanism is not yet certain.

Circular RNAs (circRNAs), a group of non-coding RNAs with covalently closed-loop structures, are generated by back-splicing of eukaryotic exons without 5’-cap and 3’-polyA and are specifically expressed in tissues and cells [6]. Many studies have demonstrated that circRNAs are involved in the process of CI/R development, including circOGDH [7], circMEMO1 [8], and circCDC14A [9]. These circRNAs act as biomarkers in the CI/R process or regulate neuronal viability, apoptosis, inflammation and oxidative stress processes. circPUM1 is an important member of the circRNA family, which has been shown to promote the development of cancers such as liver cancer and lung adenocarcinoma [10,11]. According to the circRNA expression profile in ischemic stroke, circPUM1 expression is elevated in the brain of middle cerebral artery occlusion (MCAO) rats, suggesting that it may take part in CIR injury development [12]. However, the definite function and regulatory mechanism of circPUM1 in CIR-induced neuronal injury remain to be further figured out.

CircRNAs often act as endogenous competing RNAs for microRNAs (miRNAs) and mediate post-transcriptional expression of downstream mRNAs to regulate disease progression [13]. miRNAs are a class of endogenous noncoding RNAs controlling gene expression by recognizing homologous sequences and interfering with epigenetic, transcriptional, or translational processes [14]. Studies have demonstrated the important role of miRNAs in the development of CI/R [15–17]. MiR-340-5p is an extensively explored miRNA that is linked with CIR injury. Xu C *et al*. report that miR-340-5p motivates angiogenesis of brain microvascular endothelial cells during OGD/R [18]. Tu X *et al*. discover that miR-340-5p mediates CIR injury by targeting the APPL1/LKB1/AMPK pathway [19]. However, it is unclear whether miR-340-5p is adsorbed by circRNAs in CI/R injury and thus affects the development of CI/R.

This study explored the function and underlying mechanism of circPUM1 in CIR-induced neuronal injury. We hypothesized that circPUM1 affects the process of CIR-induced neuronal injury by regulating the miR-340-5p/DDX5 axis and verified our hypothesis by *in vivo* and *in vitro* experiments. These data provide potential therapeutic targets and strong data support for future clinical treatment of CI/R injury.

## 2. Materials and methods

### 2.1. Transient MCAO (tMCAO) model establishment

Forty adult male C57/BL6 mice (18–25 g; 8 weeks old) were from Hunan Provincial Center for Disease Control and Prevention. Female mice were not applied because of the influence of estrogen. Mice were housed in standard laboratory rearing facilities at (25 ± 2)°C, 50%–60% humidity, 12-h light cycle. All mice had free access to food and purified water. The tMCAO model establishment was conducted as set forth [20]. Briefly, deep anesthetization of mice was conducted by 1–2% isoflurane oxygen/nitrous oxide mixture at a ratio of 30% and 69% through a face mask; the body temperature was maintained at (37 ± 0.3)°C on a small animal heating platform. Exposure of the left common carotid as well as the external and internal carotid arteries was conducted, and a silicone-coated 6–0 suture was routed from the stump of the external to the internal carotid artery until reaching the lumen of the middle cerebral artery. The distance from the arterial bifurcation to the middle cerebral artery was 10 ± 0.5 mm. A Laser Doppler flowmetry (Moore Instruments, UK) was applied to determine if occlusion was successfully achieved. After 60 min of occlusion, the filament was removed for reperfusion. The same procedure was done for sham-operated mice with the exception that the suture was routed along the internal carotid artery before being immediately withdrawn. After 24-h reperfusion, mice were scored for neurological function. Subsequently, mice were euthanized by inhalation of excess CO_2_ and brain tissue specimens were collected for subsequent analysis. Animal care and method procedures were authorized by the Animal Ethics Committee of Dalian Municipal Central Hospital (Approval Number SYXK2016-017WAC20).

### 2.2. Lentivirus injection

shRNA lentiviral vector targeting circPUM1 (shRNA-circPUM1) and the negative control lentiviral vector (shRNA-NC) were from GenePharma. Fourteen days before tMCAO, an injection of lentiviral vector (2 μl, 1 × 10^9^ TU/ml) into the left ventricle was done. The injection coordinates were as follows: AP, −0.3 mm; lateral, 1.0 mm; and ventral, 2.2 mm.

### 2.3. Neurological score

In the light of the Zea-Longa score, the neurological deficit was graded by two investigators in a blinded manner. 0 point, no symptoms of neurological deficit; 1 point, the rat cannot fully extend the contralateral forelimb; 2 points, the body turns to the hemiplegia side when walking; 3 points, the body leans to the hemiplegia side when walking; 4 points, the rats lose consciousness.

### 2.4. Brain water content (BW)

BW was measured by the wet/dry method [21]. Briefly, the wet weight (WW) of brain specimens was calculated by an electronic analytical balance (APX-60, Denver Instrument, NY), while the dry weight (DW) was recorded after drying in an oven at 100°C for 72 h. BM = [(ww – dw)/ww] × 100%.

### 2.5. Terminal deoxynucleotidyl transferase-mediated dUTP-biotin nick end labeling (TUNEL) and Nissl staining

Detection of brain tissue apoptotic cells was conducted by the One-Step TUNEL Apoptosis Assay kit (Roche) as set forth [22]. Briefly, brain tissues were fixed in 4% paraformaldehyde and prepared into 4 μm paraffin sections. After deparaffinization and hydration, the paraffin sections were incubated with proteinase K, and reacted with a mixture of fluorescently labeled dUTP solution and TdT enzyme. As a positive control, the sections were incubated with DNase I prior to the fluorescent labeling procedure. As a negative control, dUTP was applied. Subsequently, the sections were treated with diaminobenzidine and counterstained with 4’,6-diamidino-2-phenylindole (DAPI; Sigma-Aldrich; Merck KGaA). Then, the sections were dehydrated with gradient ethanol series, treated with xylene, and mounted with neutral balsam. For Nissl staining, the sections were stained with cresyl violet acetate, followed by dehydration, clearance, and microscopical analysis [23].

### 2.6. Immunofluorescence

After washing the slides with 0.01 mol/L phosphate-buffered saline, Triton X-100 solution was added and 0.3% H_2_O_2_ was supplemented to block the endogenous peroxidase. The sections were incubated with anti-GFAP (G3893, MilliporeSigma), and Iba-1 (MABN92, Sigma-Aldrich) overnight, and with the secondary antibody (#4409; CST) for 2 h. DAPI staining was applied to identify nuclei and a fluorescence microscope (Olympus BX51-DP70) to observe images [24,25].

### 2.7. Cell culture and transfection

Culture of human neuroblastoma cells SH-SY5Y cells (American Type Culture Collection, Rockville, USA) were in Dulbecco’s Modified Eagle Medium (Gibco, USA) covering 10% Fetal bovine serum (Gibco, USA). Transfection of miR-340-5p-mimic/inhibitor, mimic/inhibitor-negative control (NC), small interfering RNA or overexpression plasmids targeting circPUM1 and DDX5 (si/oe-PUM1/DDX5/NC) was done in cells using Lipofectamine™ 2000 Transfection Reagent (Invitrogen; Thermo Fisher Scientific, Inc). Reverse transcription quantitative polymerase chain reaction (RT-qPCR) was adopted to verify the transfection efficacy. Subsequently, SH-SY5Y cells were subjected to OGD/R treatment as set forth [26].

### 2.8. Cell counting kit (CCK)-8 detection of cell proliferation

Cells (2 × 10^3^ cells/mL, 100 μL per well) were seeded in 96-well microplates, with 5 replicate wells in each group. The adherent cells were added with CCK-8 reagent (10 μL) to each well and reacted for 2 h to determine the optical density (OD) value at 450 nm.

### 2.9. Cytotoxicity assay of lactate dehydrogenase (LDH)

Determination of cytotoxicity was implemented by an LDH assay kit (Beyotime, China). Cell supernatants were transferred to 96-well plates to detect LDH release by detecting the absorbance at 490 nm using an Enzyme-linked immunosorbent assay (ELISA) reader (SpectraMax® M5, Molecular Devices, USA) [27].

### 2.10. ELISA

ELISA was applied to detect the concentrations of IL-1β, TNF-α and IL-6 in tissues and cells [28]. The procedure was implemented in the light of the instructions of the kit (Nanjing Jiancheng Bioengineering Institute). After the reaction was terminated, the absorbance at 450 nm was read.

### 2.11. Flow cytometry

Detection of apoptosis was implemented by the Annexin V-fluorescein isothiocyanate (FITC) Apoptosis Detection Kit (DOJINDO) in the light of the manufacturer’s protocol. Briefly, cells were resuspended in Annexin V-FITC binding buffer, added with Annexin V-FITC (5 μL), centrifuged, and resuspended in Annexin V-FITC binding solution. Then, 10 μL PI solution was added, after which data were acquired on a BD FACSCalibur flow cytometer in combination with BD Cell Quest software [29].

### 2.12. RT-qPCR

Total RNA extracts collected by TRIzol reagent (Invitrogen) were subjected to reverse transcription by Hairpin-it miRNAs qPCR Quantification Kit (GenePharma, Shanghai, China) and PrimeScript RT Kit (Invitrogen), respectively. ABI 7500 system (Thermo Fisher Scientific) was employed for real-time PCR. miRNA and mRNA expression levels were normalized to U6 and β-actin, respectively. The primer sequences (Sangon Biotech) are clarified in Table 1. Gene expression was assessed by the 2^−ΔΔCt^ method [30].

**Table 1.** PCR primers

Genes	Sequences
circCCDC6	Forward: 5’- AGCCGAACTAGAACAGCATCT-3’
	Reverse: 5’- TCTCCTTCTGCAAAGCCTGA-3’
miR-128-3p	Forward: 5’- TCACAGTGAACCGGTC-3’
	Reverse: 5’- CAGTGCGTGTCGTGGAGT-3’
GAPDH	Forward: 5’- CTGCCAACGTGTCAGTGGTG-3’
	Reverse: 5’- TCAGTGTAGCCCAGGATGCC-3’
U6	Forward: 5’-CGAATTTGCGTGTCATCCTT-3’
	Reverse: 5’-CGAATTTGCGTGTCATCCTT-3’

CircCCDC6, circular RNA CCDC6; miR-128-3p, microRNA-128-3p; GAPDH, glyceraldehyde-3-phosphate dehydrogenase

### 2.13. Western blot

Extraction of total proteins in tissues and cells was conducted by Radio-Immunoprecipitation assay lysis buffer (Beyotime Biotechnology, Shanghai, China), and quantification was by bicinchoninic acid method. Total protein was separated by 12% sodium dodecyl sulfate-polyacrylamide gel electrophoresis and electroblotted onto a polyvinylidene fluoride membrane. Then, the 5% nonfat milk-blocked membrane was incubated with the following primary antibodies: p-p65 (3033), p65 (8242), cleaved caspase-3 (9661) (all Cell Signaling Technology), Bax (ab32503), DDX5 (ab21696) (both Abcam), β-actin (A5441, MilliporeSigma) and horseradish peroxidase (HRP)-conjugated secondary antibody (1: 3000). ECL (Amersham Pharmacia Biotech, Little Chalfont, UK) was employed to develop signals, which were then analyzed by Image J software.

### 2.14. The luciferase activity assay

After amplification, the wild-type (WT) circPUM1 sequence covering the predicted miR-340-5p binding site or the WT 3’-untranslated region (UTR) fragment of DDX5 mRNA was cloned into the pmirGLO dual-luciferase expression vector (Promega, Madison, WI, USA) to establish reporter vectors pmirGLO-circPUM1/DDX5-WT. Mutation of the putative binding site of miR-340-5p in circPUM1 or DDX5 3’-UTR was conducted by the GeneArtTM Site-Directed Mutagenesis PLUS System (cat. no. A14604; Thermo Fisher Scientific, Inc.). The mutant (Mut) circPUM1 or DDX5 3’-UTR was cloned into the pmirGLO vector to construct the reporter vectors pmirGLO-circPUM1/DDX5-MUT. Co-transfection of the reporter vector and miR-340-5p-mimic or mimic-NC was done in SH-SY5Y cells by Lipofectamine™ 2000. Measurement of luciferase activity was conducted by a dual-luciferase reporting system (Promega).

### 2.15. RNA immunoprecipitation (RIP) assay

RIP kit (Millipore, Bedford, MA, USA) was utilized for RIP detection [31]. Briefly, cells after reacting with RIP lysis buffer were incubated with magnetic beads conjugated to human anti-Ago2 antibody. Then, immunoprecipitated RNA was isolated after proteinase K detachment, and RNA expression was checked.

### 2.16. Data analysis

All the experiments in the research were carried out with at least three biological replicates (N = 3). GraphPad Prism 9.0 (GraphPad Software) was utilized for statistical analysis. Data reported as mean ± standard deviation (SD) were compared by Student’s t-test (two groups) or one-way analysis of variance (multi-groups). *P* < 0.05 emphasized statistical difference.

## 3. Results

### 3.1. Knockdown circPUM1 aggravates CIR nerve injury

**Figure 1.**
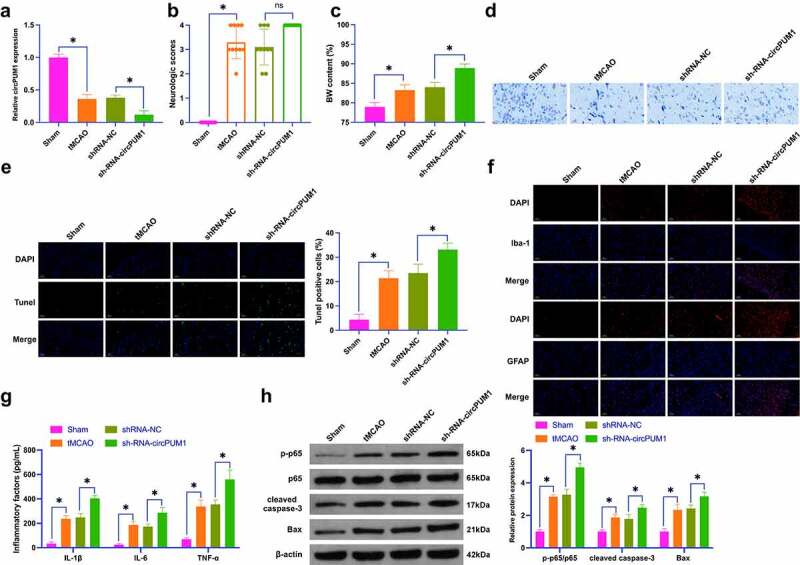
Knockdown of circPUM1 aggravates CIR neuronal injury.

To figure out circPUM1ʹs biological function in CIR, a tMCAO mouse model was established, and circPUM1 expression in tMCAO mice was knocked down by lentiviral interference. As clarified in Figure 1(a-c), tMCAO reduced circPUM1 expression, aggravated neuronal injury, and increased BM content in mice, and the injection of shRNA-circPUM1 lentivirus further promoted tMCAO-induced effects on mice. tMCAO mice were featured by a reduced number of Nissl bodies and an increased proportion of TUNEL-positive cells (Figure 1(d,e)); while knockdown of circPUM1 further worsened the pathological damage. Microglia-specific marker (Iba-1) and astrocyte-specific marker (GFAP), as well as inflammatory factors (IL-1β, IL-6 and TNF-α) were tested. In figure 1(f,g), Iba-1 and GFAP were elevated, so as these inflammatory factors in the brain tissue of tMCAO mice, while silencing circPUM1 further promoted their contents. Moreover, p-p65, cleaved caspase-3 and Bax in tMCAO mice were elevated, and knockdown of circPUM1 imposed inhibitory effects on these proteins (Figure 1(h)). These data convey that repressing circPUM1 aggravates CIR neuronal apoptosis and inflammation.

CIR neuronal injury was simulated by establishing a tMCAO mouse model, and circPUM1 was knocked down by injecting shRNA-circPUM1 lentivirus. A: RT-qPCR detection of circPUM1 in mouse brain tissue; B: Mouse neurological function score; C: Mouse brain water content; D: Nissl staining representative picture of mouse brain tissue; E: TUNEL staining representative pictures of mouse brain tissue; F: Immunofluorescence detection of Iba-1 and GFAP in mouse brain tissue; G: ELISA detection of IL-1β, IL-6 and TNF-α in mouse brain tissue; H: Western blot detection of p-p65, cleaved caspase-3, Bax; Data were presented as mean ± SD (n = 10). * *P* < 0.05.

### 3.2. Enhancing circPUM1 mitigates OGD/R-stimulated neuronal injury

**Figure 2.**
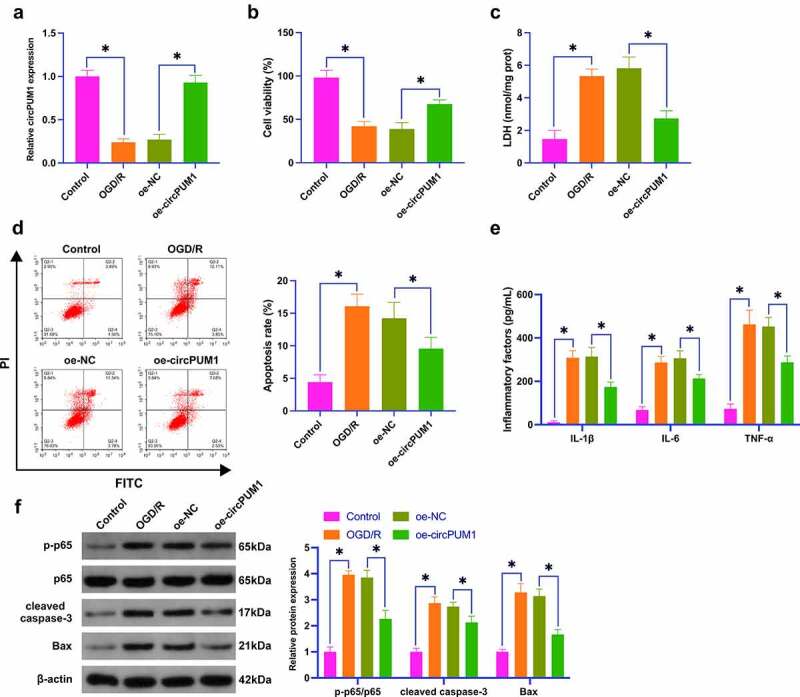
Enhancing circPUM1 mitigates OGD/R-stimulated neuronal injury.

For further learning circPUM1ʹs molecular mechanism, CIR injury was simulated *in vitro* by treating SH-SY5Y cells with OGD/R, and circPUM1 expression was overexpressed by pre-transfection of oe-circPUM1. In Figure 2(a), OGD/R treatment declined circPUM1 expression, which was mitigated by transfection of oe-circPUM1. Subsequently, detection of cell proliferation, apoptosis, LDH release and inflammation was conducted. OGD/R treatment restrained proliferation, increased the release of LDH, enhanced apoptosis and the production of inflammatory factors, as well as elevated protein expression of p-p65, cleaved caspase-3, and Bax; upon oe-circPUM1 treatment, OGD/R-induced damages to neurons were all alleviated (Figure 2(c,f)). These data conveyed that the enhancement of circPUM1 mitigates OGD/R-induced neuronal damage.

SH-SY5Y cells were treated with OGD/R to stimulate CIR neuronal injury *in vitro*, and circPUM1 in SH-SY5Y was elevated by transfection of oe-circPUM1. A: RT-qPCR detection of neuronal circPUM1; B: CCK-8 detection of neuronal viability; C: Neuronal LDH toxicity test; D: Flow cytometry detection of neuronal apoptosis; E: ELISA detection of neuronal L-1β, IL-6 and TNF-α; F: Western blot detection of p-p65, cleaved caspase-3, Bax in neurons; Data were clarified as mean ± SD (N = 3). * *P* < 0.05.

### 3.3. CircPUM1 competitively combines with miR-340-5p

**Figure 3.**
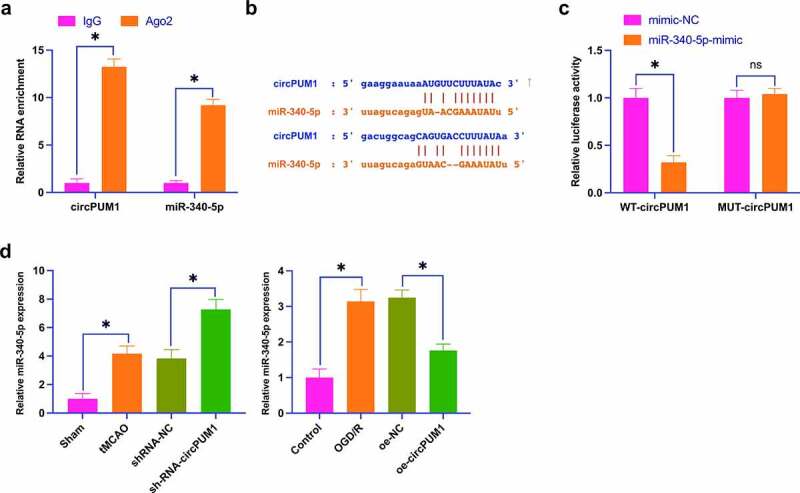
CircPUM1 competitively combines with miR-340-5p.

Next, an exploration of circPUM1ʹs downstream molecular targets was conducted. On the bioinformatics website http://starbase.sysu.edu.cn/, 20 miRNAs that had latent binding sites with circPUM1 were found. Subsequently, it was discovered that miR-340-5p had a targeted combining relation with circPUM1. In Figure 3(a), the co-transfection of WT-circPUM1 and miR-340-5p-mimic declined the luciferase activity. Figure 3(b) clarifies that circPUM1 and miR-340-5p were abundant in response to the Ago2 treatment. Meanwhile, miR-340-5p had targeted binding sites with circPUM1 at chr1:31404888-31404910[-] and chr1:31437691-31437714[-] (Figure 3(c)). Subsequently, it was examined whether miR-340-5p was targeted by circPUM1. In Figure 3(d), in both *in vivo* and *in vitro* models of CIR, miR-340-5p expression was elevated, while repression or elevation of circPUM1 motivated and restrained miR-340-5p, respectively. These data clarify that circPUM1 competitively combines with miR-340-5p.

A: The luciferase activity assay verification of the targeting link between miR-340-5p and circPUM1; B: RIP experiment verification of the combining link between miR-340-5p and circPUM1; C: Bioinformatics website prediction of latent binding sites of miR-340-5p and circPUM1; D: RT-qPCR detection of miR-340-5p expression; Data were clarified as mean ± SD (AB, N = 3; D, n = 3/10). * *P* < 0.05.

### 3.4. Elevation of miR-340-5p aggravates neuronal injury induced by OGD/R

**Figure 4.**
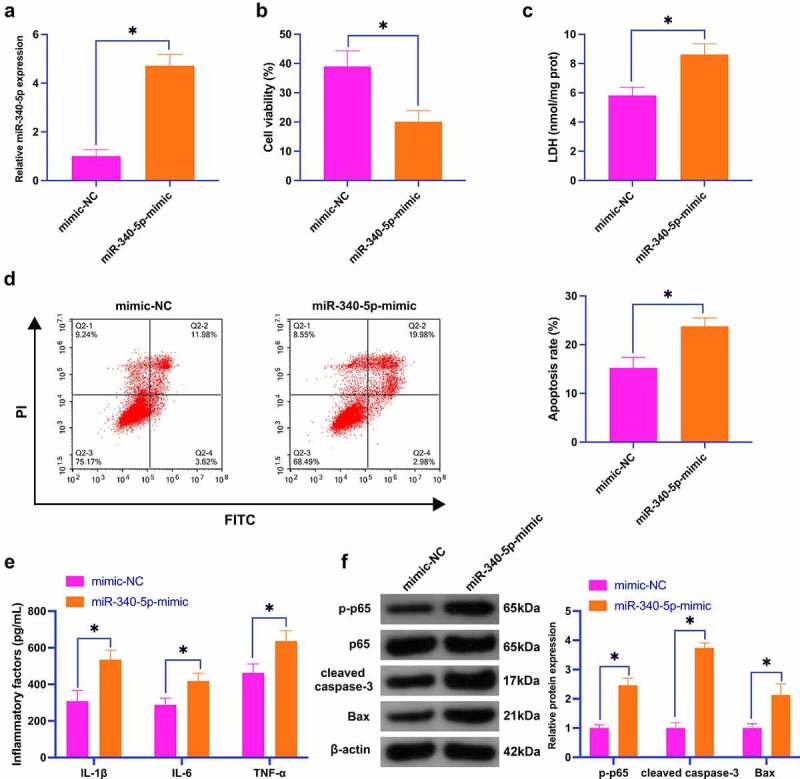
Elevation of miR-340-5p aggravates OGD/R-induced neuronal injury.

Subsequently, it was miR-340-5p-mimic transfected into SH-SY5Y cells to figure out miR-340-5p’s biological function. As clarified in Figure 4(a), transfection of miR-340-5p enhanced miR-340-5p expression in cells. Functional experiments displayed that transfection of miR-340-5p-mimic restrained cell proliferation and anti-apoptosis, increased LDH leakage and the release of inflammatory factors, and induced the protein expression of phosphorylated p65, cleaved caspase-3 and Bax (Figure 4(b-f)). These data illustrate that elevation of miR-340-5p aggravates OGD/R-induced neural damage.

MiR-340-5p in SH-SY5Y cells was elevated by transfection of miR-340-5p-mimic. A: RT-qPCR detection of neuronal miR-340-5p; B: CCK-8 detection of neuronal viability; C: Neuronal LDH toxicity test; D: Flow cytometry detection of neuronal apoptosis; E: ELISA detection of neuronal L-1β, IL-6 and TNF-α; F: Western blot detection of p-p65, cleaved caspase-3, Bax in neurons; Data were clarified as mean ± SD (N = 3). * *P* < 0.05.

### 3.5. CircPUM1 impacts OGD/R-conditioned neuronal injury by controlling miR-340-5p

**Figure 5.**
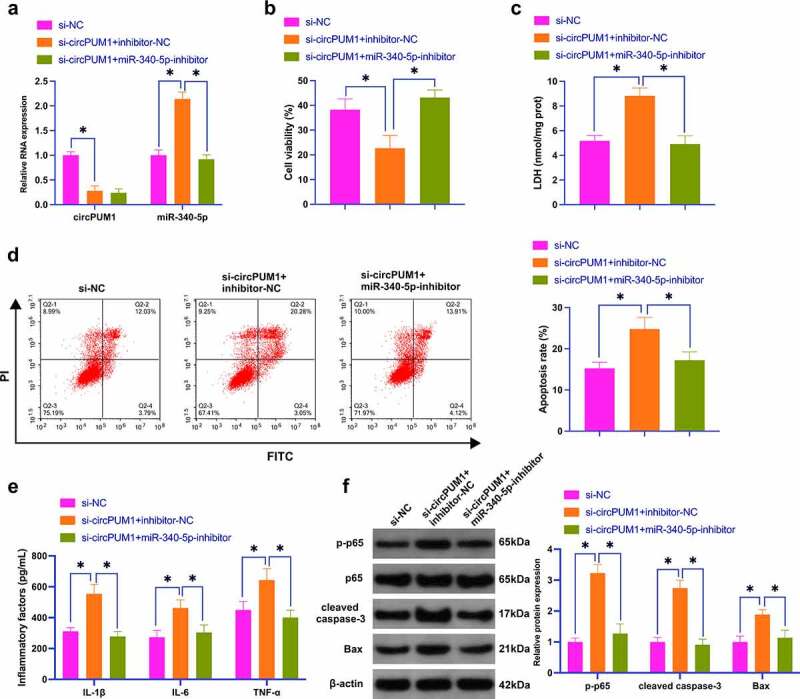
CircPUM1 impacts OGD/R-induced neuronal injury by controlling miR-340-5p.

To figure out whether circPUM1 functions in controlling OGD/R-conditioned neuronal injury through miR-340-5p, si-circPUM1 and miR-340-5p-inhibitor were co-transfected into SH-SY5Y cells. In Figure 5(a), si-circPUM1 declined circPUM1 expression but elevated miR-340-5p expression, while further treatment with miR-340-5p-inhibitor had no effect on circPUM1, but refrained miR-340-5p expression. Functional experiments displayed that the protection of si-circPUM1 against OGD/R-conditioned neuronal injury was mitigated when miR-340-5p-inhibitor was co-transfected (Figure 5(b-f)). This exhibits that circPUM1 relieves OGD/R-conditioned neuronal injury by controlling miR-340-5p.

The link between circPUM1 and miR-340-5p in OGD/R-induced neuronal injury was explored by co-transfection of si-circPUM1 and miR-340-5p-inhibitor. A: RT-qPCR detection of neuronal miR-340-5p and circPUM1; B: CCK-8 detection of neuronal viability; C: Neuronal LDH toxicity test; D: Flow cytometry detection of neuronal apoptosis; E: ELISA detection of neuronal L-1β, IL-6 and TNF-α; F: Western blot detection of p-p65, cleaved caspase-3, Bax in neurons; Data were clarified as mean ± SD (N = 3). * *P* < 0.05.

### 3.6. MiR-340-5p targets DDX5

**Figure 6.**
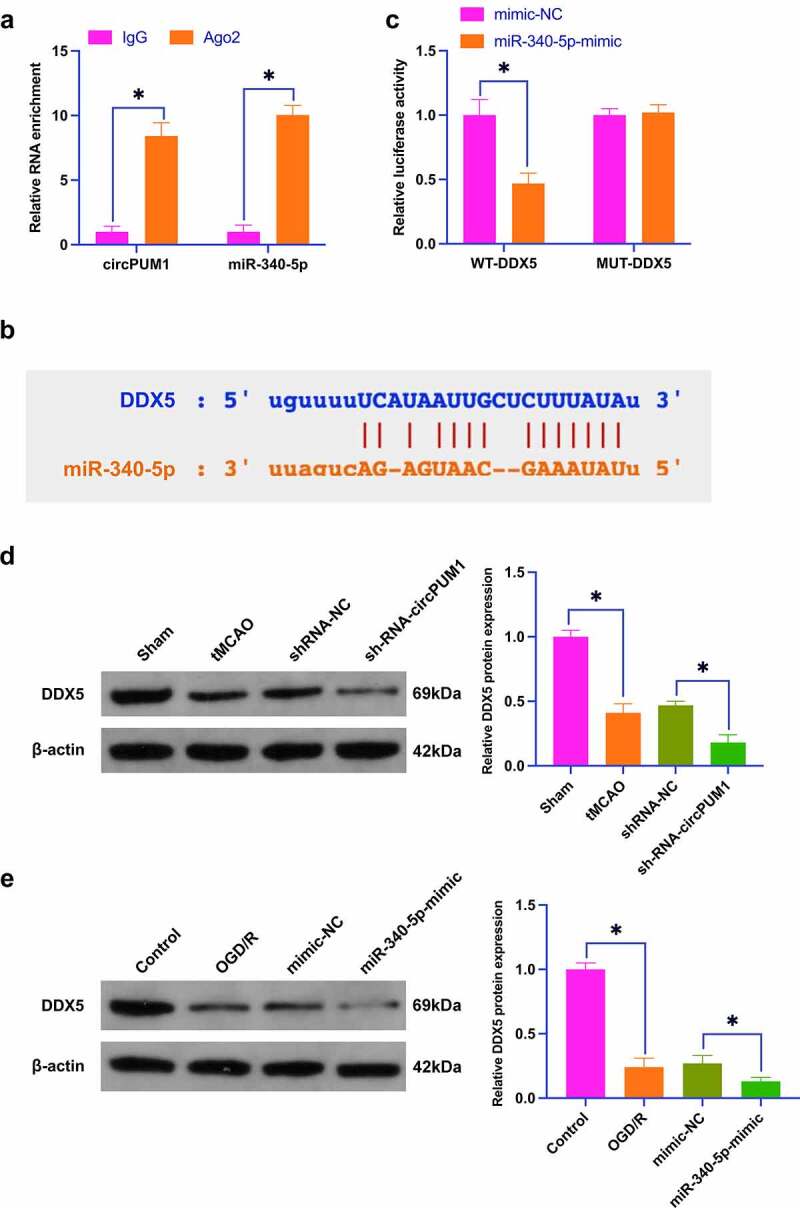
MiR-340-5p targets DDX5.

On the bioinformatics website https://starbase.sysu.edu.cn/, 10 latent target genes of miR-340-5p were searched, among which DDX5 was determined. In Figure 6(a), the co-transfection of WT-DDX5 and miR-340-5p-mimic declined the luciferase activity. Figure 6(b) clarifies that DDX5 and miR-340-5p could be enriched by Ago2 rather than IgG. Meanwhile, a binding site exhibited at chr17:62495992-62495998[-] for DDX5 and miR-340-5p (Figure 6(c)). Subsequently, an examination of the regulatory impacts of circPUM1 and miR-340-5p on DDX5 was implemented. In CIR mice, it was discovered that DDX5 expression was decreased which was further suppressed if si-circPUM1 was administrated (Figure 6(d)). Moreover, OGD/R treatment decreased DDX5 expression, and this effect was motivated by miR-340-5p-mimic (Figure 6(e)). This conveys that miR-340-5p targets DDX5.

A: The luciferase activity assay verification of the targeting link between miR-340-5p and DDX5; B: RIP experiment verification of the combining link between miR-340-5p and DDX5; C: Bioinformatics website prediction of latent binding sites of DDX5 and circPUM1; D: Western blot detection of the effect of circPUM1 knockdown on DDX5 in CIR mice; E: Western blot detection of the effect of elevation of miR-340-5p on DDX5 in SH-SY5Y cells induced by OGD/R; Data were clarified as mean ± SD (ABE, N = 3; D, n = 10). * *P* < 0.05.

### 3.7. CircPUM1 mitigates OGD/R-induced neuronal injury by controlling miR-340-5p/DDX5 axis

**Figure 7.**
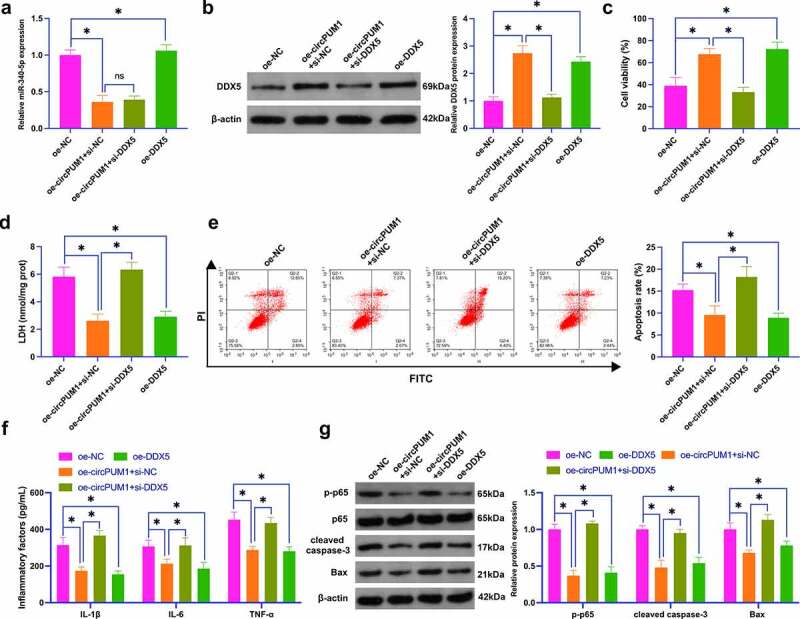
CircPUM1 mitigates OGD/R-induced neuronal injury by controlling miR-340-5p/DDX5 axis.

Next, DDX5ʹs functions in OGD/R-induced neuronal injury and the link between circPUM1 and DDX5 were figured out. Additionally, oe/si-DDX5 and oe-circPUM1 were co-transfected into SH-SY5Y cells. As clarified in Figure 7(a), oe-circPUM1 reduced miR-340-5p, while oe/si-DDX5 had no effect on miR-340-5p. Moreover, oe-circPUM1/-DDX5 enhanced DDX5 expression, while oe-circPUM1ʹs functions could be turned around by si-DDX5 (Figure 7(b)). Functional experiments clarified that oe-DDX5 protected neurons against OGD/R-induced injury, but si-DDX5 prevented oe-circPUM1-induced protection on OGD/R-injured neurons (Figure 7(c-g)). These data illustrate that circPUM1 mitigates OGD/R-induced neuronal injury by controlling miR-340-5p/DDX5 axis.

The function of DDX5 in OGD/R-conditioned neuronal injury was explored by transfection of oe-DDX5, and the link between circPUM1 and DDX5 in OGD/R-induced neuronal injury was explored by co-transfection of oe-circPUM1 and si-DDX5. A: RT-qPCR detection of neuronal miR-340-5p; B: Western blot detection of neuronal DDX5; C: CCK-8 detection of neuronal viability; D: Neuronal LDH toxicity test; E: Flow cytometry detection of neuronal apoptosis; F: ELISA detection of neuronal L-1β, IL-6 and TNF-α; G: Western blot detection of p-p65, cleaved caspase-3, Bax in neurons; Data were clarified as mean ± SD (N = 3). * *P* < 0.05.

## 4. Discussion

Ischemic stroke is a major illness of the brain with severe CIR damage, which ultimately causes neuronal cell death and disruption of neuronal circuits in the brain and spinal cord [32]. As a group of crucial regulators, circRNAs have been confirmed to take on a key role in controlling CIR damage [33]. In the research, it was discovered that circPUM1 was elevated in the tMCAO mouse model as well as in OGD/R-treated SH-SY5Y cells, and repression of circPUM1 could aggravate CIR neuronal apoptosis and inflammation *in vivo*. Furthermore, *in vitro* experimental results clarified that elevation of circPUM1 lessened OGD/R-induced neuronal damage by targeting the miR-340-5p/DDX5 axis.

Several studies have illustrated that circRNA take on a crucial role in CIR damage. Circ_002664 is elevated in OGD/R-induced neurons and motivates neuronal apoptosis by directly targeting the miR-182-5p/Herpud1 pathway [34]. circHECTD1 is elevated in OGD/R-induced neurons and MCAO mice, and knockdown of circHECTD1 relieves CIR-induced neuronal damage via the miR-133b/TRAF3 pathway [35]. Circ_008018 is upregulated in MCAO rat brain, and repressing circPUM1 mitigates CIR-induced brain tissue damage and neurological deficit by targeting miR-99a [36]. In this research, it was discovered a novel circRNA, circPUM1, was downregulated after CIR injury, whereas repressing circPUM1 aggravated neuronal apoptosis, inflammation and cytotoxicity. It was testified that the downstream molecular pathway of circPUM1 was the miR-340-5p/DDX5. CircPUM1 elevated DDX5 expression by binding with miR-340-5p, thereby controlling neuronal apoptosis and inflammation. In addition, previous studies have confirmed the positive role of circPUM1 in cancer development [10,11], the results of this study further reveal the mechanism of circPUM1 in ameliorating CI/R injury, and this molecular regulation pathway may provide new insights into the development of targeted drugs for CIR injury.

Numerous studies have clarified multiple miRNAs participate in CIR-induced neuronal damage. For example, elevating miRNA-27a attenuates CIR-induced oxidative stress, nerve damage, and inflammatory responses via activating the PI3K/AKT/mTOR pathway [37]. Elevation of miR-211 protects against CIR injury by refraining neuronal apoptosis [38]. MiR-30c exerts neuroprotection in CIR rats by targeting SOX9 [39]. MiR-340-5p is discovered to be reduced in the peripheral blood of ischemic stroke patients, suggesting that it may take part in CIR injury [40]. Zheng Yi [41] *et al*. discover that miR-340-5p expression is reduced in OGD/R-induced neurons, and enhancement of miR-340-5p alleviates OGD/R-induced neuronal damage by targeting PDCD4 to activate PI3K/Akt pathway. The results of this study further support these views.

DDX5 is a member of the DEAD-box RNA helicase family, which controls ribonucleoprotein formation [42]. DDX5 normally functions by performing as a transcriptional co-activator of several transcription factors and has been shown to motivate tumorigenesis and cancer progression [43,44]. Furthermore, under hypoxic conditions, DDX5 is elevated by miR-462 and restrains cell advancement by blocking cell cycle progression of DNA replication [45]. In the research, it was found that DDX5 expression was elevated in OGD/R-treated SH-SY5Y cells, and repression of DDX5 reversed the effect of elevation of circPUM1 on motivating CIR-induced neuronal damage and inflammatory responses. A former study illustrates DDX5 is available to restrain Ser311 phosphorylation of the p65 subunit, and knockdown of DDX5 motivates TNF-α-induced apoptosis [46]. In the present study, it was also found that knockdown of DDX5 elevated the phosphorylation of p65 and motivated inflammatory factors in SH-SY5Y cells. This indicates that the NF-κB pathway is a momentous downstream pathway regulated by DDX5.

Notably, circPUM1 is only a part of the circRNA family. In previous studies, various circRNAs, such as circOGDH, circMEMO1, and circCDC14A, have been shown to regulate CIR. Therefore, we believe that the role of the circPUM1/miR-340-5p/DDX5 axis in CIR injury is limited. However, circRNAs and their downstream molecular networks are of great significance in the development of CIR injury, so it is necessary to further reveal the functions of different circRNAs in CI/R injury, which will provide the basis for the subsequent development of drugs for the treatment of CIR injury. In the present study, no clinical study of CI/R injury was performed on circPUM1. We speculate that circPUM1/miR-340-5p/DDX5 has a similar effect in the clinic. It is necessary to explore the serum levels of circPUM1 in different periods of CIR in follow-up studies, which may serve as a new biomarker of CIR.

## 5. Conclusion

Taken together, the results of this study convey circPUM1 motivates CIR nerve injury by regulating the miR-340-5p/DDX5 axis. This finding offers brand-new insights into the mechanism by which circPUM1 regulates CIR-induced neural injury and inflammatory responses, and a latent target for the cure of CIR injury after ischemic stroke.

## Supplementary Material

Supplemental Material
