# Supplementary figures and images for: Circular RNA PUM1 performs as a competing endogenous RNA of microRNA-340-5p to mediate DEAD-box helicase 5 to mitigate cerebral ischemia-reperfusion injury
Source: Bioengineered. 2022 May 5;13(5):11564–78. doi: 10.1080/21655979.2022.2068923 (PMC9276027; doi:10.1080/21655979.2022.2068923)

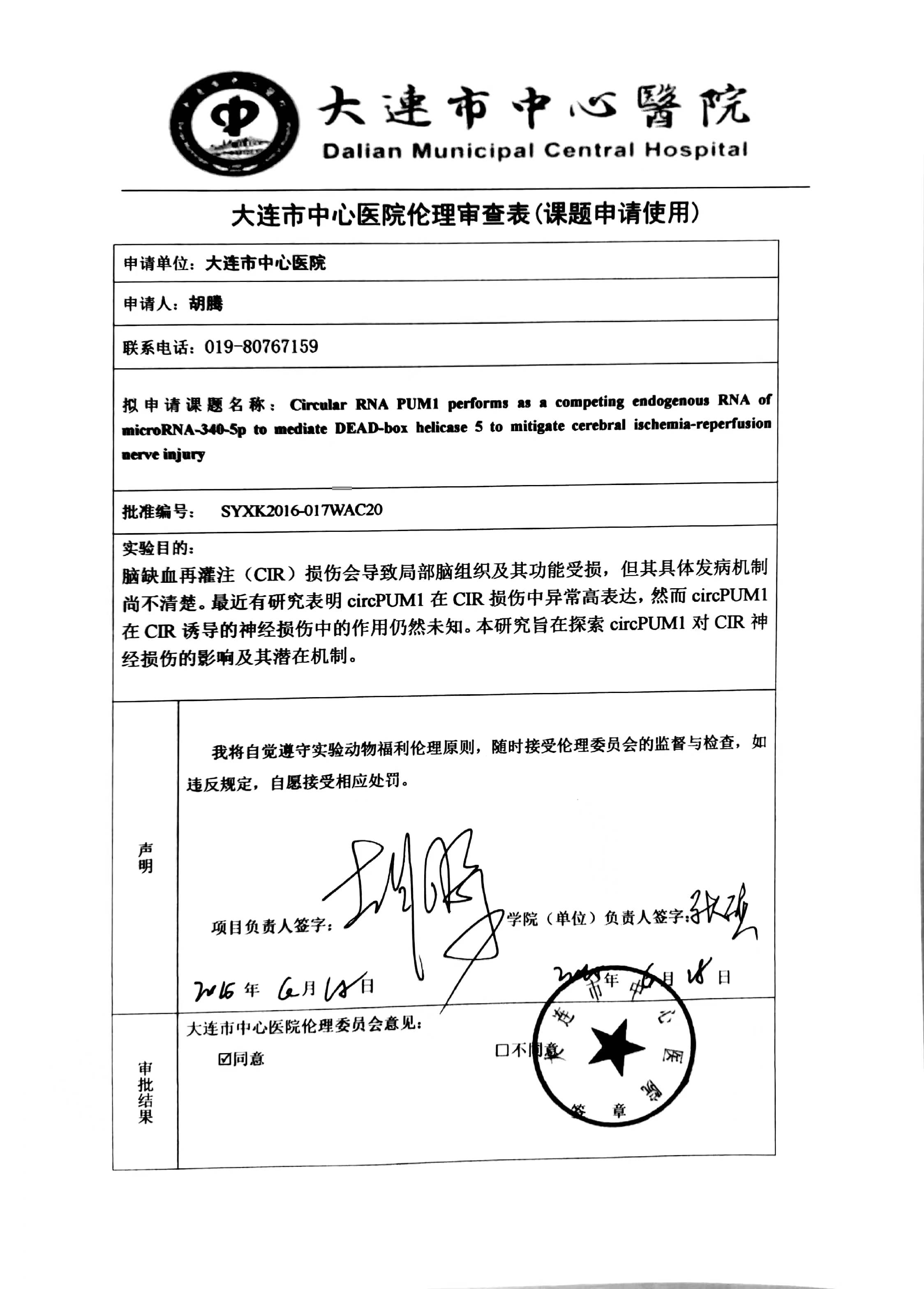

Supplement: Supplemental Material [file KBIE_A_2068923_SM3413.zip › supplementary/Ethical Approval Document.jpg]
